# Robustness of tissue oxygenation estimates by continuous wave space-resolved near infrared spectroscopy

**DOI:** 10.1117/1.JBO.28.7.075002

**Published:** 2023-07-17

**Authors:** Caterina Amendola, Davide Contini, Rebecca Re, Lorenzo Spinelli, Lorenzo Frabasile, Pietro Levoni, Alessandro Torricelli

**Affiliations:** a Politecnico di Milano, Dipartimento di Fisica, Milan, Italy.; b Istituto di Fotonica e Nanotecnologie, Consiglio Nazionale delle Ricerche, Milan, Italy

**Keywords:** near infrared spectroscopy, spatially resolved spectroscopy, tissue oxygen saturation, reduced scattering coefficient, differential pathlength factor

## Abstract

**Significance:**

Continuous wave near infrared spectroscopy (CW-NIRS) is widely exploited in clinics to estimate skeletal muscles and brain cortex oxygenation. Spatially resolved spectroscopy (SRS) is generally implemented in commercial devices. However, SRS suffers from two main limitations: the *a priori* assumption on the spectral dependence of the reduced scattering coefficient [$\mu_{s}^{\prime }(\lambda )$] and the modeling of tissue as homogeneous.

**Aim:**

We studied the accuracy and robustness of SRS NIRS. We investigated the errors in retrieving hemodynamic parameters, in particular tissue oxygen saturation ($S_{t}O_{2}$), when $\mu_{s}^{\prime }(\lambda )$ was varied from expected values, and when layered tissue was considered.

**Approach:**

We simulated hemodynamic variations mimicking real-life scenarios for skeletal muscles. Simulations were performed by exploiting the analytical solutions of the photon diffusion equation in different geometries: (1) semi-infinite homogeneous medium and constant $\mu_{s}^{\prime }(\lambda )$; (2) semi-infinite homogeneous medium and linear changes in $\mu_{s}^{\prime }(\lambda )$; (3) two-layered media with a superficial thickness $s_{1}=5$, 7.5, 10 mm and constant $\mu_{s}^{\prime }(\lambda )$. All simulated data were obtained at source-detector distances ρ=35, 40, 45 mm, and analyzed with the SRS approach to derive hemodynamic parameters (concentration of oxygenated and deoxygenated hemoglobin, total hemoglobin concentration, and tissue oxygen saturation, $S_{t}O_{2}$) and their relative error.

**Results:**

Variations in $\mu_{s}^{\prime }(\lambda )$ affect the estimated $S_{t}O_{2}$ (up to ±10%), especially if changes are different at the two wavelengths. However, the main limitation of the SRS method is the presence of a superficial layer: errors strongly larger than 20% were retrieved for the estimated $S_{t}O_{2}$ when the superficial thickness exceeds 5 mm.

**Conclusions:**

These results highlight the need for more sophisticated strategies (e.g., the use of multiple short and long distances) to reduce the influence of superficial tissues in retrieving hemodynamic parameters and warn the SRS users to be aware of the intrinsic limitation of this approach, particularly when exploited in the clinical environment.

## 1 Introduction

There is a growing number of applications at both a clinical and consumer level where near infrared spectroscopy (NIRS) is used to noninvasively monitor tissue hemodynamics in skeletal muscle or cerebral cortex. By exploiting the capability of near infrared light to penetrate deeply into biological tissues and the specific spectral features of oxygenated and deoxygenated hemoglobin, it is in fact possible, from light attenuation measurements, to noninvasively estimate the molar concentration of these chromophores in the tissue under investigation, and then retrieve tissue oxygen saturation ($S_{t}O_{2}$). This parameter can be used as a biomarker for physio-pathological conditions in the clinics^1^ or as an indicator of skeletal muscle fatigue during exercise and sports activities.^2^ Due to the diffusive nature of biological tissues, $S_{t}O_{2}$ estimate requires either discrimination of absorption and scattering contribution, as provided by the time domain (TD) NIRS or frequency domain (FD) NIRS,^3^ or the adoption of specific measurement strategy, like in the space-resolved reflectance (SRS) approach.^4^

Nowadays, the SRS approach is being used in many continuous wave (CW)- NIRS devices for clinical research and for consumer applications targeting skeletal muscle or brain hemodynamics. A recent database of available instruments can be found in Ref. 5. The characteristic feature of CW SRS NIRS is the possibility to noninvasively provide an estimate for $S_{t}O_{2}$—the so-called tissue oxygenation index or tissue saturation index—based on the spatial gradient of light attenuation detected at two wavelengths by two or three narrowly spaced detectors. The practical implementation of CW SRS NIRS brings to a simpler and cheaper device than FD NIRS or TD NIRS that can naturally provide $S_{t}O_{2}$.

However, the CW SRS NIRS approach has some basic limitations mainly related to the lack of a direct estimate of the reduced scattering coefficient $\mu_{s}^{\prime }(\lambda )$, to the heterogeneity of the medium, and to the use of simplifying assumption on modeling boundary conditions for reemitted photons.

A first fundamental assumption of the SRS method is in fact the *a priori* knowledge of the spectral dependence of $\mu_{s}^{\prime }(\lambda )$ that is typically approximated by a simple linear dependence $\mu_{s}^{\prime }(\lambda )=k(1-h\lambda )$, where k and h are empirical parameters. Actually, the exact value of k is not needed in the derivation of $S_{t}O_{2}$ (see Sec. 2 for details), whereas the value of h is crucial. To overcome the assumption on $\mu_{s}^{\prime }(\lambda )$, a broadband SRS approach can be implemented^6^^,^^7^ but that would require a more complex setup^8^ jeopardizing the compactness and simplicity of the SRS approach. Therefore, in most of the compact and wearable CW-NIRS devices, a minimum set of two wavelengths are implemented, making the assumption on $\mu_{s}^{\prime }(\lambda )$ rather critical. Moreover, during a prolonged measurement, $\mu_{s}^{\prime }(\lambda )$ is usually kept constant, whereas it has not yet been perfectly understood whether variations of the structural organization of the tissue itself can induce changes in $\mu_{s}^{\prime }(\lambda )$.^9^^,^^10^ To our knowledge, a detailed study on the effect of $\mu_{s}^{\prime }(\lambda )$ on SRS still has not been conducted, whereas this problem has been tackled in the context of TD NIRS^11^^,^^12^ or FD NIRS.^13^

A second important assumption of the SRS method is that the probed medium is spatially homogeneous, that is, the optical properties do not vary with the source-detector distance and with the depth from the surface. On the contrary, tissue homogeneity in depth is frequently violated in skeletal muscle oximetry, because of the presence of the adipose tissue over the muscle, and in cerebral oximetry, due to the layered structure of the human head (with scalp, skull, cerebrospinal fluid, gray matter, and white matter). In CW-NIRS, to tackle tissue heterogeneity, a multi-distance or tomographic scheme should be implemented, again at the cost of increased complexity of the device.^14^ Adding a short source-detector distance channel (<10  mm) to sample superficial tissue hemodynamics is a solution recommended in functional NIRS setups.^15^ However, to our knowledge, this approach has not been implemented in SRS NIRS. “Few works have investigated the effect of superficial adipose tissue on SRS results^16^^,^^17^ and the effect of a layered medium with different optical properties,^6^^,^^18^ whereas a systematic study is still missing.

An additional minor limitation of the SRS NIRS approach is the use of the so-called “zero-boundary” condition (ZBC) that proved to be less accurate for describing light propagation at tissue boundaries.^19^

This study aims to explore the accuracy and robustness of the SRS NIRS approach for estimating hemodynamic parameters, in particular the $S_{t}O_{2}$. We will first recall the basic theory behind SRS, then we will study the accuracy and the robustness of the SRS NIRS approach for the estimate of $S_{t}O_{2}$ and of the hemodynamic parameters by means of numerical simulations in the framework of the photon diffusion theory.

## 2 Theory

The SRS approach models the tissue as a semi-infinite medium with absorption coefficient $\mu_{a}$ and reduced scattering coefficient $\mu_{s}^{\prime }$, and it adopts a reflectance geometry configuration with a collimated light source and a source detector distance ρ. Under the extrapolated boundary condition (EBC), the CW reflectance (the number of photons per unit area exiting the medium) at a distance ρ is given by

$$R(\rho )=\frac{1}{4\pi }\{\begin{array}{c} z_{s}\frac{1+\mu_{\mathrm{eff}}(\rho^{2}+z_{s}^{2}{)}^{-\frac{1}{2}}}{(\rho^{2}+z_{s}^{2}{)}^{\frac{3}{2}}}e^{\left[-\mu_{\mathrm{eff}}(\rho^{2}+z_{s}^{2}{)}^{\frac{1}{2}}\right]}+\\ +z_{b}\frac{1+\mu_{\mathrm{eff}}{\left(\rho^{2}+z_{b}^{2}\right)}^{-\frac{1}{2}}}{(\rho^{2}+z_{b}^{2}{)}^{3/2}}e^{\left[-\mu_{\mathrm{eff}}(\rho^{2}+z_{b}^{2}{)}^{\frac{1}{2}}\right]} \end{array}\}\;,\;$$ (1)

where $z_{s}=1/\mu_{s}^{\prime }$, $z_{b}=2z_{e}+z_{s}$, $z_{e}=2BD$ is the extrapolation distance, $D={\left(3\mu_{s}^{\prime }\right)}^{-1}$ is the diffusion coefficient, $\mu_{\mathrm{eff}}=(\frac{\mu_{a}}{D}{)}^{\frac{1}{2}}$ is the effective attenuation coefficient, and B is a parameter accounting for refractive index mismatch at the medium surface.^20^

Both the original derivation of the SRS method^4^ and the recent developments^7^ employ the simpler ZBC obtained by setting $z_{e}=0$. Despite the EBC solution for the reflectance is known to be more accurate than the ZBC solution, it was demonstrated that for the SRS approach, the use of the EBC yields no significant gain over the ZBC at the cost of an increased complexity in the computation.^7^ By adopting the ZBC and applying the common approximation $\rho \gg z_{s}$, we have

$$R(\rho )≅\frac{z_{s}}{2\pi }\rho^{-3}(1+\mu_{\mathrm{eff}}\rho )e^{-\mu_{\mathrm{eff}}\rho }.$$ (2)

Light attenuation is defined as A(ρ)=−ln(R(ρ)). The SRS method derives $\mu_{\mathrm{eff}}$ from ∂A/∂ρ, the spatial derivative of A as follows:

$$\mu_{\mathrm{eff}}=\frac{1}{2}\{(\frac{\partial A}{\partial \rho }-\frac{3}{\rho })+{\left[{\left(\frac{\partial A}{\partial \rho }-\frac{3}{\rho }\right)}^{2}+\frac{4}{\rho }(\frac{\partial A}{\partial \rho }-\frac{3}{\rho })\right]}^{\frac{1}{2}}\}.$$ (3)

Note that often in the implementation of the SRS method, the additional approximation $\mu_{\mathrm{eff}}\;\rho \gg 1$ is commonly used, leading to a simpler formula for $\mu_{\mathrm{eff}}$

$$\mu_{\mathrm{eff}}≅(\frac{\partial A}{\partial \rho }-\frac{2}{\rho }).$$ (4)

However, there is no significant reduction in complexity or computation time using Eq. (4) with respect to Eq. (3), therefore in the following we will always use Eq. (3).

Combining Eq. (3) with the definition of $\mu_{\mathrm{eff}}$, we can derive the absorption coefficient, typically expressed as a function of wavelength (λ) and experiment time (T)

$$\mu_{a}(\lambda ,T)=\frac{\mu_{\mathrm{eff}}^{2}(\lambda ,T)}{3\mu_{s}^{\prime }(\lambda ,T)}.$$ (5)

The *a priori* knowledge of $\mu_{s}^{\prime }$ is needed to derive $\mu_{a}$. In the classical SRS approach, a constant value for $\mu_{s}^{\prime }$ is used, typically approximated as^4^

$$\mu_{s}^{\prime }(\lambda ,T)≅\mu_{s}^{\prime }(\lambda )≅k(1-h\lambda ).$$ (6)

If we are only interested in deriving $S_{t}O_{2}=O_{2}\mathrm{Hb}/(O_{2}\mathrm{Hb}+\mathrm{HHb})$, then we can simply assume h and leave k unknown. By the Beer’s law and knowing the specific absorption coefficients $\varepsilon_{O_{2}\mathrm{Hb}}(\lambda )$ and $\varepsilon_{\mathrm{HHb}}(\lambda )$,^21^ Eqs. (5) and (6) yield $k\mu_{a}(\lambda ,T)=\frac{\mu_{\mathrm{eff}}^{2}(\lambda ,T)}{3(1-h\lambda )}$, from which we can derive $kO_{2}\mathrm{Hb}(T)$ and kHHb(T). Therefore, the parameter k simplifies in the derivation of $S_{t}O_{2}$.

In the Biomedical Optics community, the spectral feature of the reduced scattering coefficient is typically approximated by means of an empirical power law derived from Mie’s theory as

$$\mu_{s}^{\prime }(\lambda )≅a{\left(\frac{\lambda_{0}}{\lambda }\right)}^{b},$$ (7)

where $\lambda_{0}$ is a reference wavelength, $a=\mu_{s}^{\prime }(\lambda_{0})$ is related to the volume density of equivalent scatterers, and b is the so-called scatter power coefficient that depends on the size of the equivalent scatterers.^22^

The linear approximation for $\mu_{s}^{\prime }$ employed in the SRS approach can be interpreted as a first order approximation of Eq. (7). Using the classic Taylor expansion method for $\mu_{s}^{\prime }(\lambda )$ around a given $\lambda_{1}$, one obtains in fact

$$\mu_{s}^{\prime }(\lambda )=a{\left(\frac{\lambda_{0}}{\lambda_{1}}\right)}^{b}≅(1+b)(1-\frac{b}{(1+b)\lambda_{1}}\lambda )=k(1-h\lambda ).$$ (8)

## 3 Material and Methods

### 3.1 Simulations

**Fig. 1:**
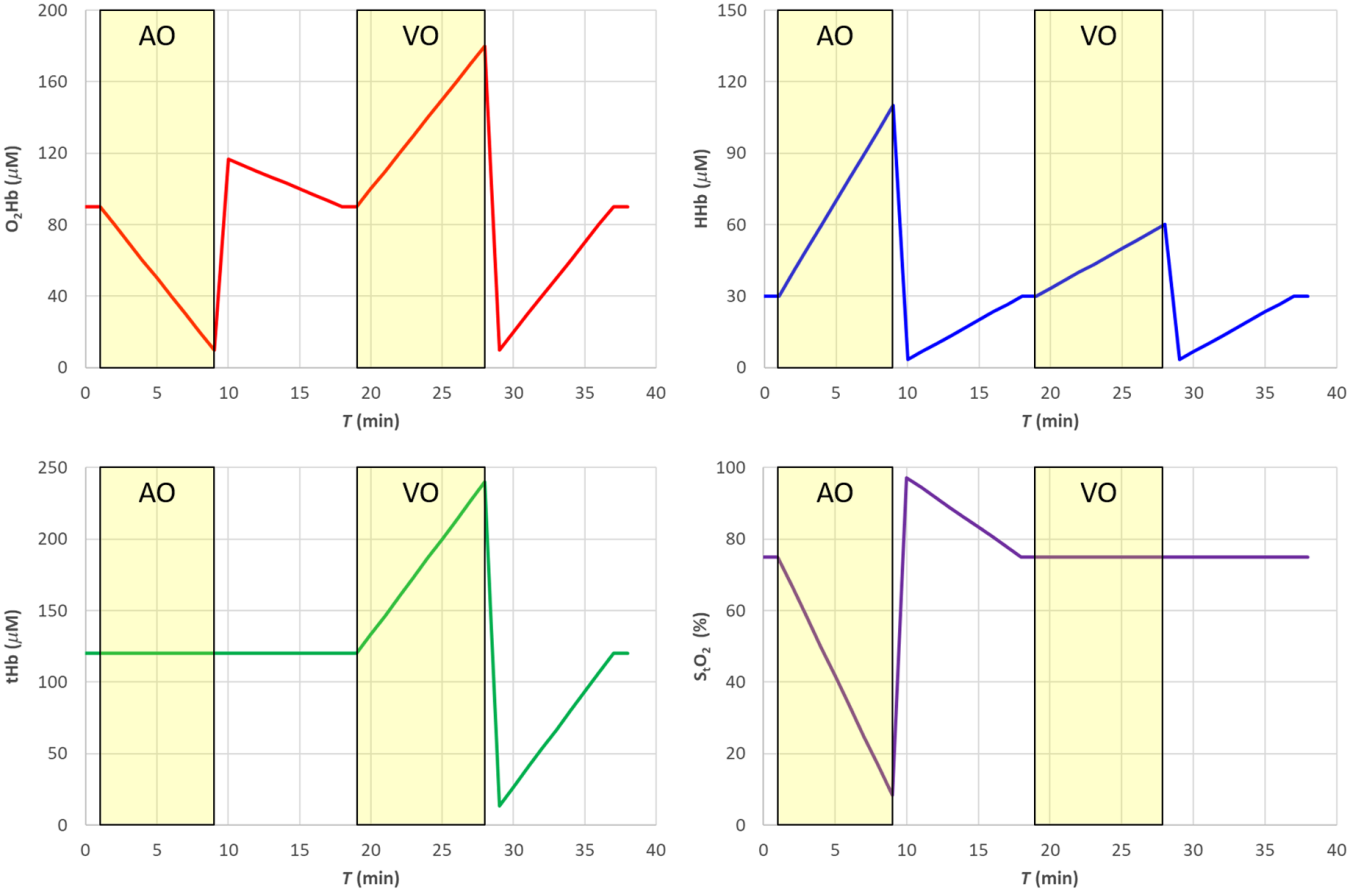
The nominal values of used in the simulations of Secs. 4.1–4.3 and 4.5. The shaded yellow areas represent the AO and VO tests.

We simulated a hemodynamic experiment (total duration 39 min, with time points every 1 min) with changes in the concentration of $O_{2}\mathrm{Hb}$ and HHb mimicking realistic conditions for a skeletal muscle (e.g., deoxygenation following arterial cuff occlusion, hyperemic peak after cuff release, venous occlusion), as shown in Fig. 1. Baseline values (T=0  min) were ${\left(O_{2}\mathrm{Hb}\right)}_{0}=90\;\mu M$, ${\left(\mathrm{HHb}\right)}_{0}=30\;\mu M$, ${\left(\mathrm{tHb}\right)}_{0}={\left(O_{2}\mathrm{Hb}\right)}_{0}+{\left(\mathrm{HHb}\right)}_{0}=120\;\mu M$, and $(S_{t}O_{2}{)}_{0}=75\%$.

In a first set of simulations, from T=1  min to T=9  min, we mimicked an arterial occlusion (AO), with tHb kept constant at baseline value and $S_{t}O_{2}$ progressively reducing to 8.3%, followed from T=10  min to T=18  min by the hyperemic reperfusion and return to baseline values. Then, from T=19  min to T=28  min, we mimicked a venous occlusion (VO), with $S_{t}O_{2}$ kept constant at baseline value and a linear increase of tHb up to 240  μM, followed by a reversed change mimicking a reduction of perfusion with constant $S_{t}O_{2}$ followed by a return to baseline values.

**Fig. 2:**
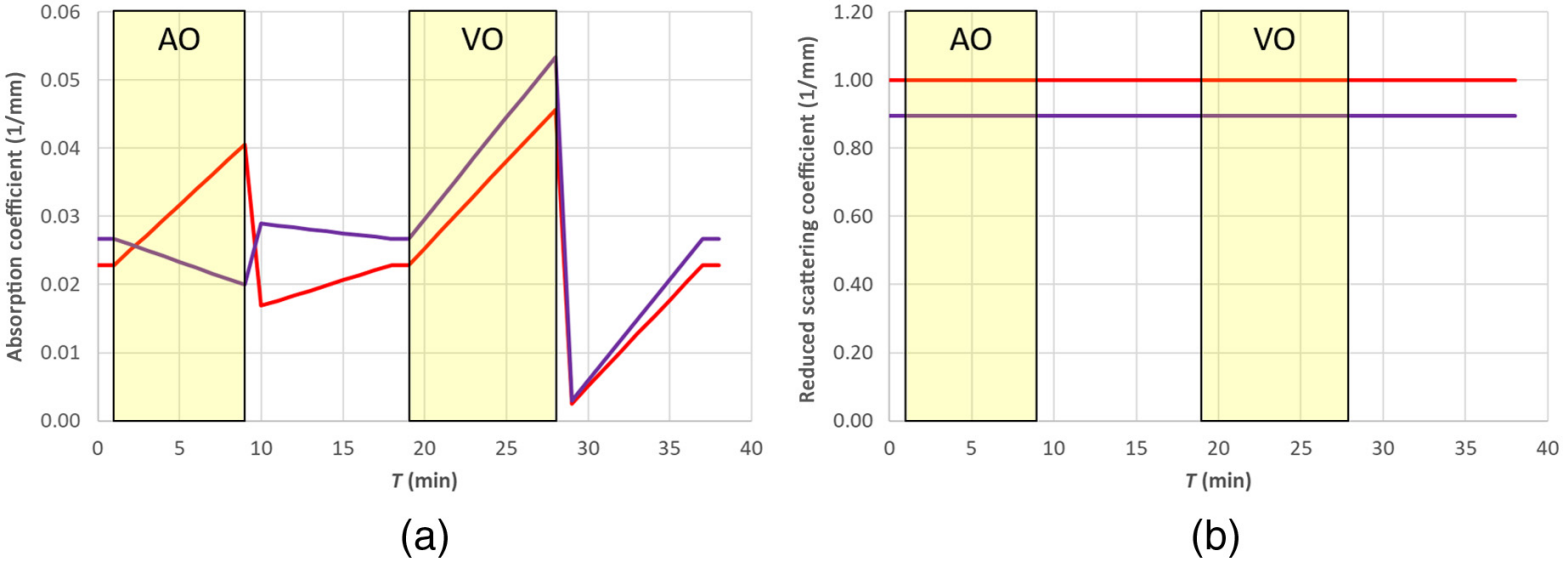
The nominal values of (a) $\mu_{a}(\lambda ,T)$ and (b) $\mu_{s}^{\prime }(\lambda ,T)$ used in the simulations of Secs. 4.1–4.3 and 4.5. Red 760 nm, purple 850 nm. The shaded yellow areas represent the AO and VO tests.

The absorption coefficients at 760 and 850 nm (see Fig. 2) were then derived as linear combination of the concentrations of $O_{2}\mathrm{Hb}$ and HHb by means of the Beer’s law with the knowledge of the specific absorption coefficients $\varepsilon_{O_{2}\mathrm{Hb}}(\lambda )$ and $\varepsilon_{\mathrm{HHb}}(\lambda )$.^21^

The baseline reduced scattering coefficient was calculated by means of Eq. (7), assuming $a=1\;{\mathrm{mm}}^{-1}$, b=1, and $\lambda_{0}=760\;\mathrm{nm}$, resulting in 1.0 and $0.89\;{\mathrm{mm}}^{-1}$ at 760 and 850 nm, respectively.

Once the optical properties were obtained, we used analytical solutions of the photon diffusion equation in the EBC approximation in different geometries to calculate the CW reflectance at three source-detector distances (ρ=35, 40, 45 mm) at wavelengths 760 and 850 nm.^20^^,^^23^

In the first set of simulations, a semi-infinite medium was considered, and the reduced scattering coefficient was kept constant at the baseline value (see Secs. 4.1–4.3).

In a second simulation, a semi-infinite medium was considered, and the reduced scattering coefficient was linearly varied with respect to the initial value during the experiment time (see Sec. 4.4), whereas the hemodynamic parameters were maintained constant.

Finally, a two-layer medium was considered^23^ (two-layer cylinder with different thicknesses of the upper layer: $s_{1}=5$, 7.5, 10 mm and total thickness $s_{0}=90\;\mathrm{mm}$), and hemodynamic changes were applied either to the upper or to the lower medium layer while keeping the hemodynamic parameters of the other layer constant at the baseline values (see Sec. 4.5).

A schematic of the main parameters used in the forward simulations is reported in Table 1.

**Table 1:** Details on the main parameters used in the forward simulations and in the SRS approach.

Section	Medium	Layer thickness		Forward				SRS approach			Figure
		Upper	Lower	$\mu_{a}^{\text{Upper}}$	$\mu_{a}^{\text{Lower}}$	$\mu_{s}^{\prime \text{Upper}}$	$\mu_{s}^{\prime \text{Lower}}$	$\mu_{s}^{\prime }$	$\mu_{\mathrm{eff}}$	$\mu_{a}$	
Sec. 4.1	Semi-infinite	∞	NA	Variable Fig. 1 (left)	NA	Constant Fig. 1 (right) Eq. (7): $\lambda_{0}=760\;\mathrm{nm}$ $a=1\;{\mathrm{mm}}^{-1}$, b=1	NA	Exact Eq. (7): $\lambda_{0}=760\;\mathrm{nm}$ $a=1\;{\mathrm{mm}}^{-1}$, b=1	Eq. (3) With A(ρ)=−ln(R(ρ)) $\frac{\partial A}{\partial \rho }≅\frac{A(\rho_{L})-A(\rho_{S})}{\rho_{L}-\rho_{S}}$ $\frac{1}{\rho }≅\frac{1}{\rho_{S}}≅\frac{\ln(\frac{\rho_{L}}{\rho_{S}})}{\rho_{L}-\rho_{S}}$	Eq. (5)	Figs. 3, 4 Filled circle (•)
Sec. 4.2	Semi-infinite							Approximate Eq. (8): $\lambda_{0}=760\;\mathrm{nm}$ $\lambda_{1}=805\;\mathrm{nm}$ $a=1\;{\mathrm{mm}}^{-1}$, b=1			Data not shown
Sec. 4.3	Semi-infinite							Wrong a Eq. (7): $\lambda_{0}=760\;\mathrm{nm}$ $\lambda_{1}=805\;\mathrm{nm}$ $a=0.8/1.2\;{\mathrm{mm}}^{-1}$, b=1			Figs. 3, 4 empty diamond (⋄) +20%, cross (X) –20%
								Wrong b Eq. (7): $\lambda_{0}=760\;\mathrm{nm}$ $\lambda_{1}=805\;\mathrm{nm}$ $a=1\;{\mathrm{mm}}^{-1}$, b=0.8/1.2			Figs. 5, 6 empty diamond (⋄) +20%, cross (X) –20%
								Wrong h Eq. (8): $\lambda_{0}=760\;\mathrm{nm}$ $\lambda_{1}=805\;\mathrm{nm}$ $h=0.8/1.2\;{\mathrm{mm}}^{-1}$, k=1			Data not shown
								Wrong k Eq. (8): $\lambda_{0}=760\;\mathrm{nm}$ $\lambda_{1}=805\;\mathrm{nm}$ $h=1\;{\mathrm{mm}}^{-1}$, k=0.8/1.2			Data not shown
Sec. 4.4	Semi-infinite	∞	NA	Constant Fig. 7 (left)	NA	Variable Fig. 7 (right)	NA	Constant $\mu_{s}^{\prime }(\lambda )=\mu_{s0^{\prime }}(\lambda )$			Figs. 8, 9
Sec. 4.5	Two-layer	5, 7.5, 10 mm	85, 82.5, 80 mm	Constant Fig. 7 (left)	Variable Fig. 1 (left)	Constant Fig. 1 (right) Eq. (7): $\lambda_{0}=760\;\mathrm{nm}$ $a=1\;{\mathrm{mm}}^{-1}$, b=1	Constant Fig. 1 (right) Eq. (7): $\lambda_{0}=760\;\mathrm{nm}$ $a=1\;{\mathrm{mm}}^{-1}$, b=1	Exact Eq. (7): $\lambda_{0}=760\;\mathrm{nm}$ $a=1\;{\mathrm{mm}}^{-1}$, b=1			Figs. 10, 11
Sec. 4.5				Variable Fig. 1 (left)	Constant Fig. 7 (left)						Figs. 12, 13

NA, not applicable.

### 3.2 Data Analysis

From the simulated CW reflectance at different source detector distances R(ρ), we derived the spatial derivative of the attenuation by means of central finite differences as $\frac{\partial A}{\partial \rho }≅\frac{A(\rho_{L})-A(\rho_{S})}{\rho_{L}-\rho_{S}}$, with $\rho_{S}$ and $\rho_{L}$ the shortest and longest source detector distances, respectively.

As clearly described by,^14^ in the practical implementation when using two source detector distances $\rho_{S}$ and $\rho_{L}=\rho_{S}+\delta$, with $\delta \ll \rho_{S}$, the numerical approximation $\frac{1}{\rho }≅\frac{1}{\rho_{S}}≅\frac{\ln(\frac{\rho_{L}}{\rho_{S}})}{\rho_{L}-\rho_{S}}$ can be used in Eq. (3).

Applying Eq. (5), it was then possible to derive the estimate of $\mu_{a}(\lambda ,T)$, provided the assumption on $\mu_{s}^{\prime }(\lambda )$ given by Eq. (7) or Eq. (8). Then, the hemodynamic parameters were derived by inverting the Beer’s law.

A schematic of the parameters employed in the data analysis is also reported in Table 1.

## 4 Results

### 4.1 Case 1: Semi-Infinite Medium and Exact Values for the Reduced Scattering Coefficient

For a preliminary test, we simulated CW reflectance data for a semi-infinite medium with EBC, and we estimated the hemodynamic parameters by means of the SRS approach assuming the exact knowledge of the reduced scattering coefficient [Eq. (5)]. In this way, the discrepancy between estimated and nominal values can be attributed to the use of the ZBC and to the simplifying approximation $\rho \gg z_{s}$.

**Fig. 3:**
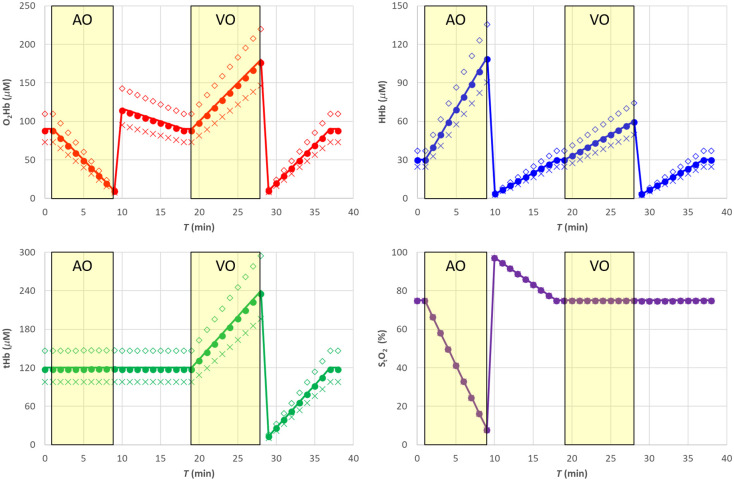
SRS results for semi-infinite homogeneous medium. Filled circle (•) exact $\mu_{s}^{\prime }(\lambda )$, empty diamond (⋄) 20% overestimation of parameter a, cross (X) 20% underestimation of parameter a. The solid line in each panel is the nominal value. The shaded yellow areas represent the AO and VO tests.

**Fig. 4:**
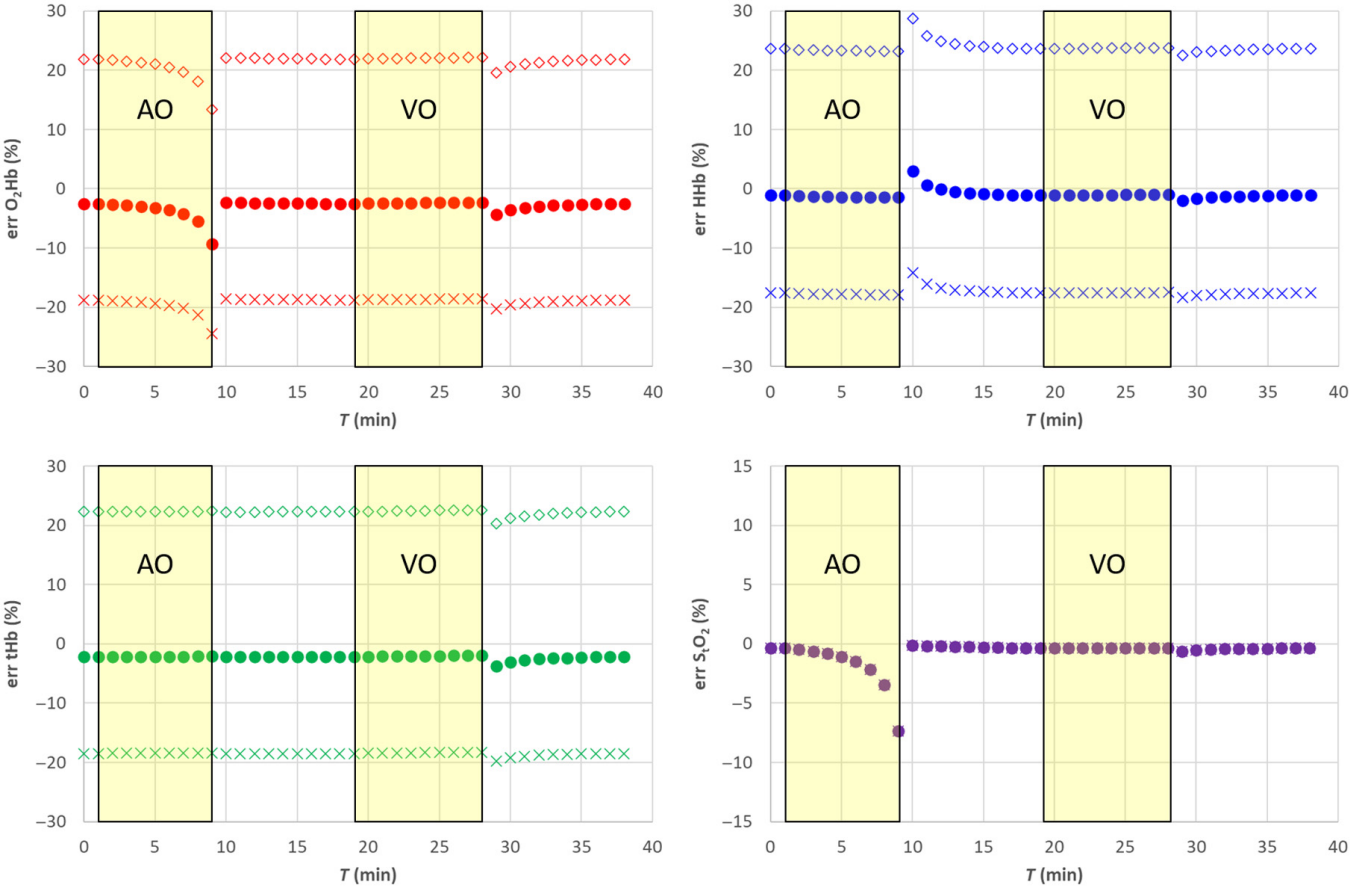
Relative error for the SRS results for semi-infinite homogeneous medium. Filled circle (•) exact $\mu_{s}^{\prime }(\lambda )$, empty diamond (⋄) 20% overestimation of parameter a, cross (X) 20% underestimation of parameter a. The shaded yellow areas represent the AO and VO tests.

In Fig. 3, the filled circle data points (•) report the results for the hemodynamic parameters. Figure 4 shows the corresponding relative error with respect to the nominal values. The estimates of the hemodynamic parameters are quite good, being the modulus of the relative error for $S_{t}O_{2}$ lower than 1% for $S_{t}O_{2}>40\%$, whereas for lower $S_{t}O_{2}$, values it increases to about 8%. Errors for $O_{2}\mathrm{Hb}$, HHb, and tHb stay within about 2%, 1% and 2%, respectively, for $S_{t}O_{2}>40\%$ while reaching about 8%, 4% and 3% for lower $S_{t}O_{2}$ values.

### 4.2 Case 2: Semi-Infinite Medium and Approximated Formula for the Reduced Scattering Coefficient

The same data of Sec. 4.1 were used assuming the linear approximation for the reduced scattering coefficient described in Eq. (8). We chose $\lambda_{1}=805\;\mathrm{nm}$, average of 760 and 850 nm, yielding for the baseline value $k=1.89\;{\mathrm{mm}}^{-1}$ and $h=6.21\;10^{4}\;{\mathrm{nm}}^{-1}$. This approximation introduces a very small relative error (about −0.3%) on the reduced scattering coefficient at both wavelengths, therefore the performance of the SRS approach is not appreciably changed (data not shown).

### 4.3 Case 3: Semi-Infinite Medium and Over/Underestimation of the Reduced Scattering Coefficient

The same data of Sec. 4.1 were used assuming a wrong value for the reduced scattering coefficient $\mu_{s}^{\prime }(\lambda )$. Considering Eqs. (7) and (8), the values for $\mu_{s}^{\prime }(\lambda )$ can be set in different ways.

Assuming a wrong value for the parameter a, it is equivalent in assuming a wrong value for parameter k [see eq. (8)]. This affects both $O_{2}\mathrm{Hb}$ and HHb and consequently also tHb as shown in Figs. 3 and 4. A 20% overestimation (underestimation) error on parameter a adds approximately an additional 20% overestimation (underestimation) on $O_{2}\mathrm{Hb}$, HHb, and tHb. However, as expected, the error on a has no influence on $S_{t}O_{2}$.

**Fig. 5:**
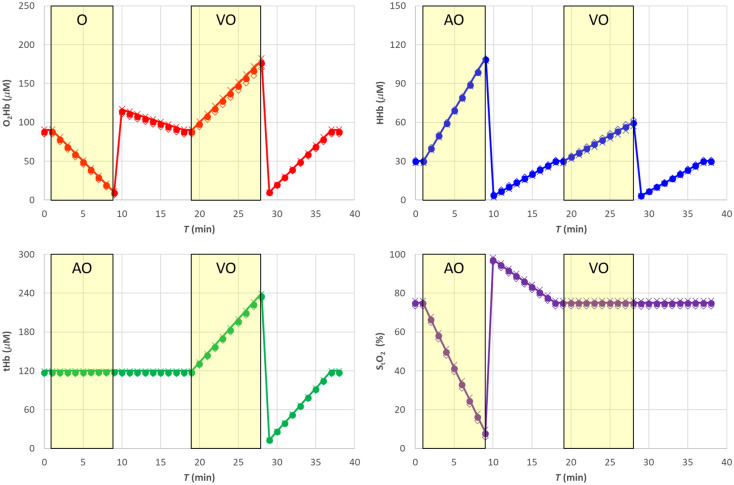
SRS results for semi-infinite homogeneous medium. Filled circle (•) exact $\mu_{s}^{\prime }(\lambda )$, empty diamond (⋄) 20% overestimation of parameter b, cross (X) 20% underestimation of parameter b. The solid line in each panel is the nominal value. The shaded yellow areas represent the AO and VO tests.

**Fig. 6:**
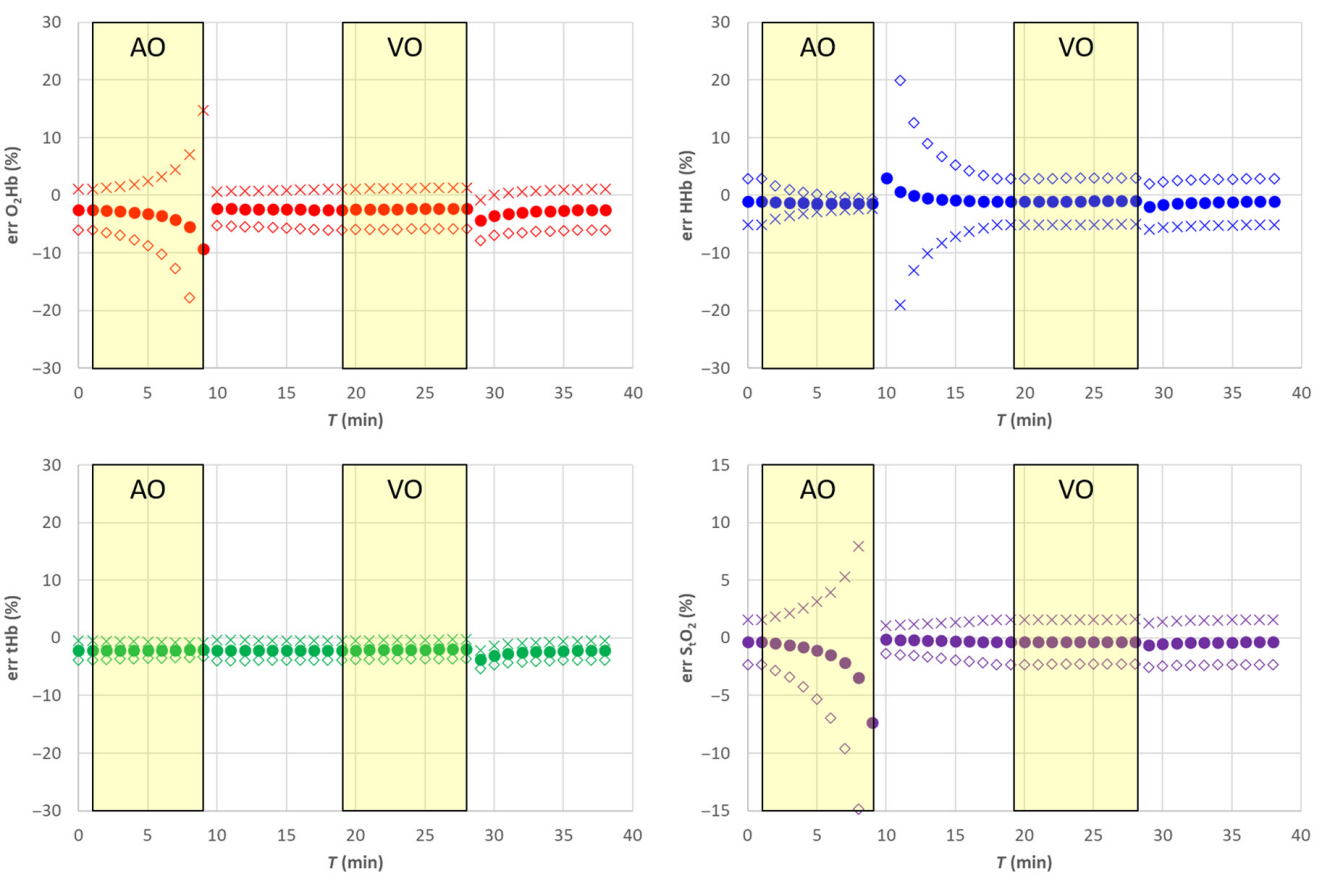
Relative error for the SRS results for semi-infinite homogeneous medium. Filled circle (•) exact $\mu_{s}^{\prime }(\lambda )$, empty diamond (⋄) 20% overestimation of parameter b, cross (X) 20% underestimation of parameter b. The shaded yellow areas represent the AO and VO tests.

Assuming a wrong value for parameter b, it is equivalent to simultaneously changing parameter k and parameter h. A 20% underestimation (overestimation) in parameter b yields approximately a 10% overestimation (underestimation) error in parameter k and parameter h. Overall, that has a smaller influence on all hemodynamic parameters including $S_{t}O_{2}$, $O_{2}\mathrm{Hb}$, and tHb, whereas the error on HHb is negligible, as shown in Figs. 5 and 6.

In the framework of the SRS approach, the assumption on parameter h is crucial. Assuming a wrong value for parameter h only (±20%) has large effect on all hemodynamic (data not shown). The modulus of the errors for $O_{2}\mathrm{Hb}$ and tHb is larger than 20%. A smaller error (10%) is obtained for HHb. The error on $S_{t}O_{2}$ is around 5% for $S_{t}O_{2}>40\%$ but rapidly increases above 10% for $S_{t}O_{2}<40\%$.

### 4.4 Case 4: Semi-Infinite Medium and Temporal Changes of the Reduced Scattering Coefficient

Now, we suppose that the reduced scattering coefficient is not constant, but it changes with the experiment time T according to the relation $\mu_{s}^{\prime }(\lambda ,T)=\mu_{s0}^{\prime }(\lambda )+\Delta \mu_{s}^{\prime }(\lambda ,T)$, where $\mu_{s0}^{\prime }(\lambda )$ is a constant baseline value and $\Delta \mu_{s}^{\prime }(\lambda ,T)$ is the change with respect to the baseline. It is straightforward to derive that if in the SRS approach, we assume the fixed value $\mu_{s}^{\prime }(\lambda )=\mu_{s0}^{\prime }(\lambda )$, then a wrong value for the absorption coefficient is obtained: $\mu_{a}^{*}(\lambda ,T)=\gamma \mu_{a}(\lambda ,T)$, with $\gamma =1+\Delta \mu_{s}^{\prime }(\lambda ,T)/\mu_{s0}^{\prime }(\lambda )=1+\delta (\lambda ,T)$. If the percentage change δ(λ,T) is the same at all wavelengths, the effect is similar as introducing an error in parameter a or k, therefore we expect no changes in $S_{t}O_{2}$, whereas we can obviously have changes in $O_{2}\mathrm{Hb}$, HHb, and tHb. Conversely, if $\delta (\lambda_{1},T)\neq \delta (\lambda_{2},T)$, the estimate of $S_{t}O_{2}$ will be affected since that is equivalent to introducing an error in parameter b or h.

**Fig. 7:**
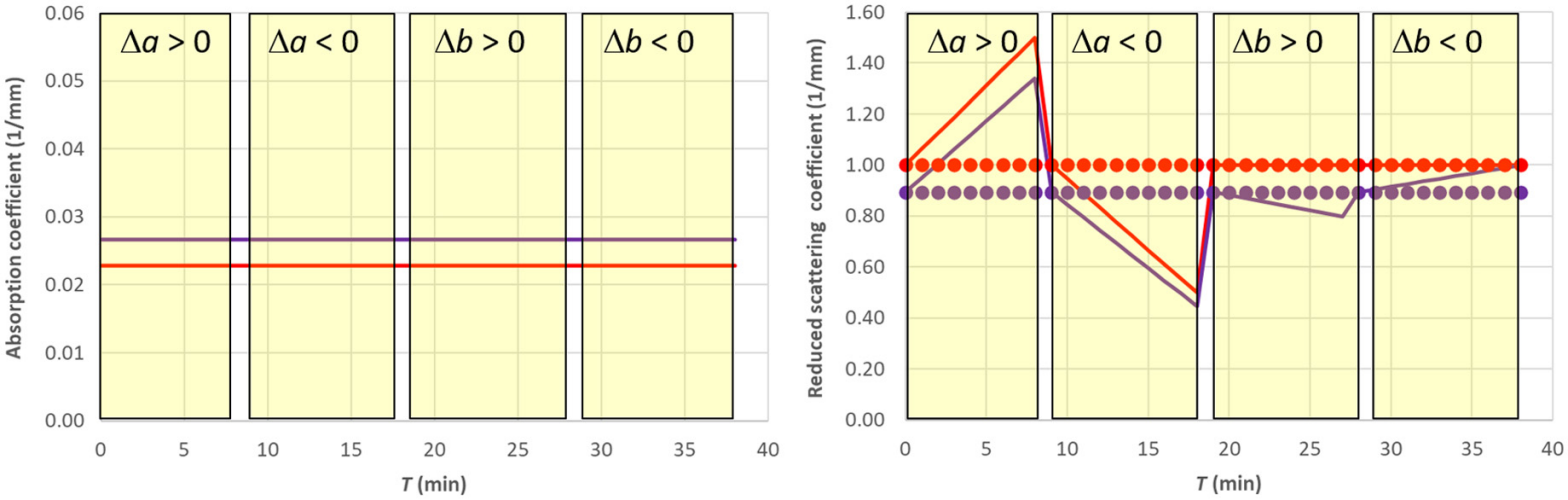
The solid lines show the nominal values of $\mu_{a}(\lambda ,T)$ and $\mu_{s}^{\prime }(\lambda ,T)$ used in the simulations of Sec. 4.4. Red 760 nm, purple 850 nm. The shaded yellow areas represent the different changes applied to the scattering parameters. In the right panel, the points show the values used in the SRS estimation.

After simulating a scenario with constant hemodynamic parameters and variable reduced scattering coefficient $\mu_{s}^{\prime }(\lambda ,T)$, we have estimated the hemodynamic parameters by assuming a constant $\mu_{s0}^{\prime }(\lambda )$, as shown in Fig. 7. In particular, in the forward simulation, linear changes with respect to the baseline were applied keeping fixed parameter b and varying parameter a up to ±50%. Then, we changed parameter b up to ±100% while keeping constant parameter a. Note that after Eq. (7), in this last case $\mu_{s}^{\prime }(\lambda_{0},T)=\mu_{s0}^{\prime }(\lambda_{0})$, the reduced scattering coefficient at 760 nm is kept constant.

**Fig. 8:**
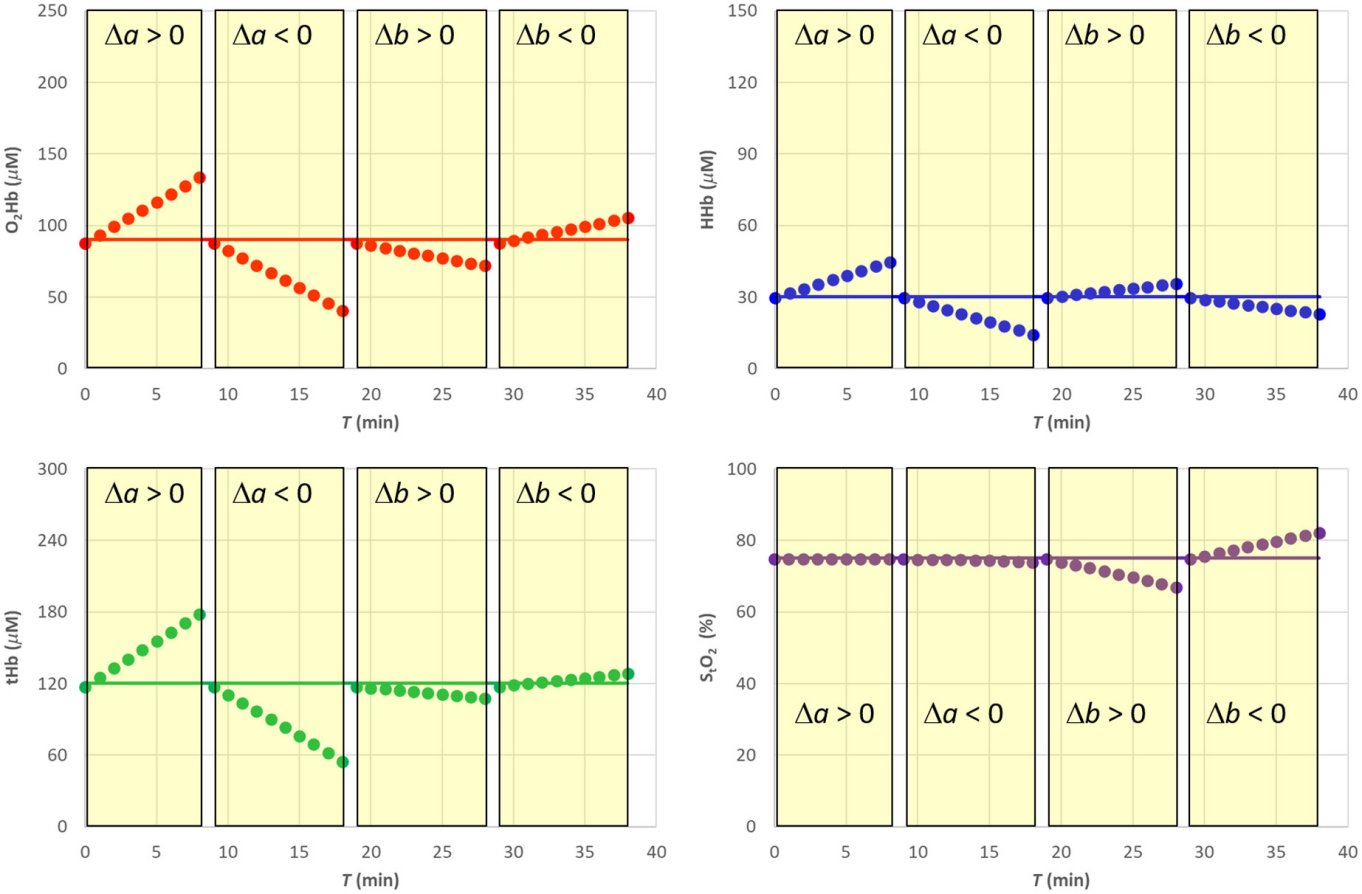
SRS results for semi-infinite homogeneous medium with variable reduced scattering when using a constant reduced scattering for SRS estimation.

**Fig. 9:**
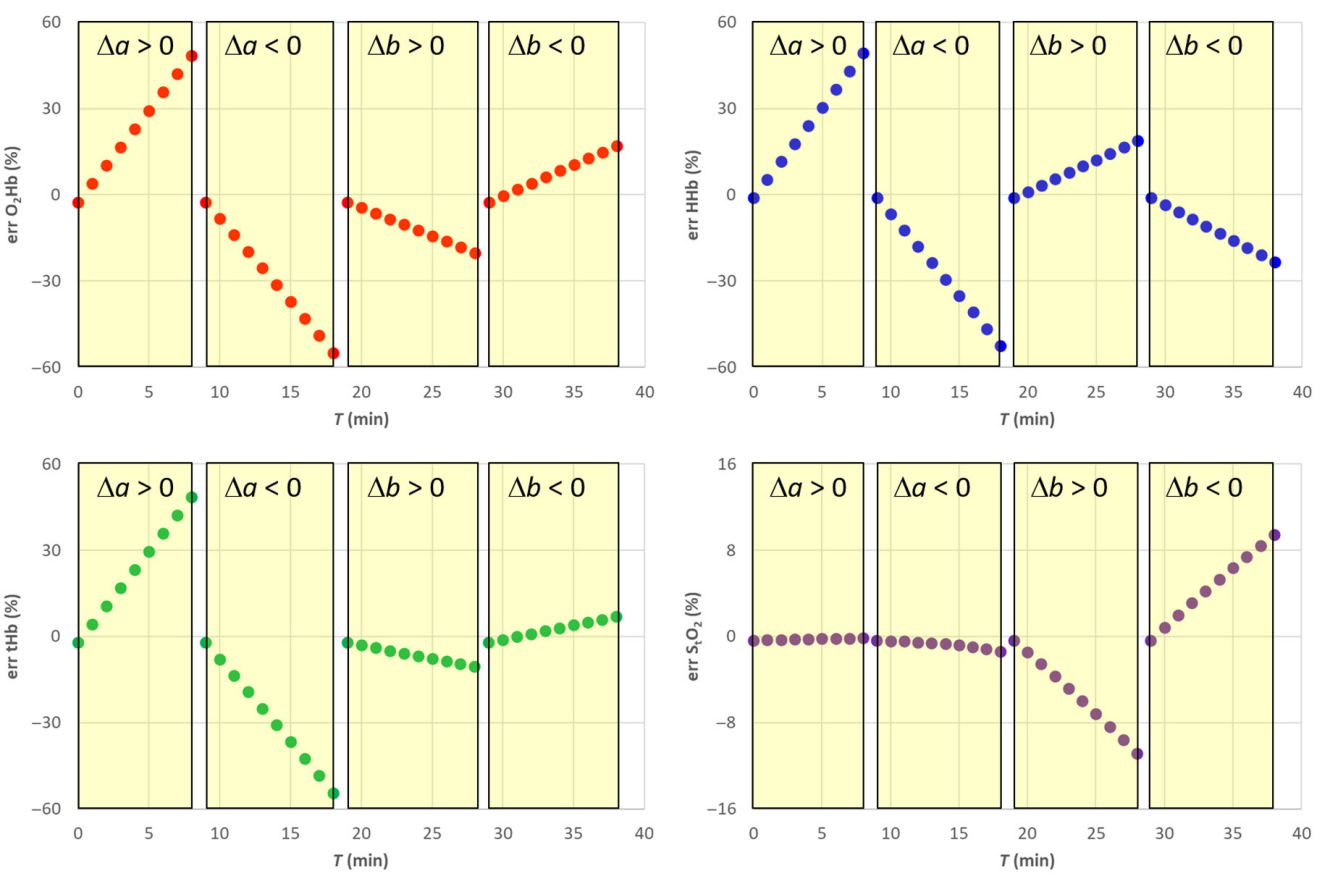
Relative error for the SRS results for semi-infinite homogeneous medium with variable reduced scattering when using a constant reduced scattering for SRS estimation.

As expected, changes in parameter a yield significant errors in the estimates of $O_{2}\mathrm{Hb}$, HHb, and tHb (see Figs. 8 and 9). However, as expected, it does not affect the estimate of $S_{t}O_{2}$. Conversely, when parameter b changes, we get an error up to about ±10% in the estimate of $S_{t}O_{2}$.

### 4.5 Case 5: Effect of the Presence of a Superficial Layer

The assumption of homogeneous medium is expected to be violated in most biomedical applications, such as skeletal muscle oximetry (due to the presence of an adipose tissue layer overlying the muscle) or cerebral oximetry (due to the presence of scalp, skull, and cerebrospinal fluid).

**Fig. 10:**
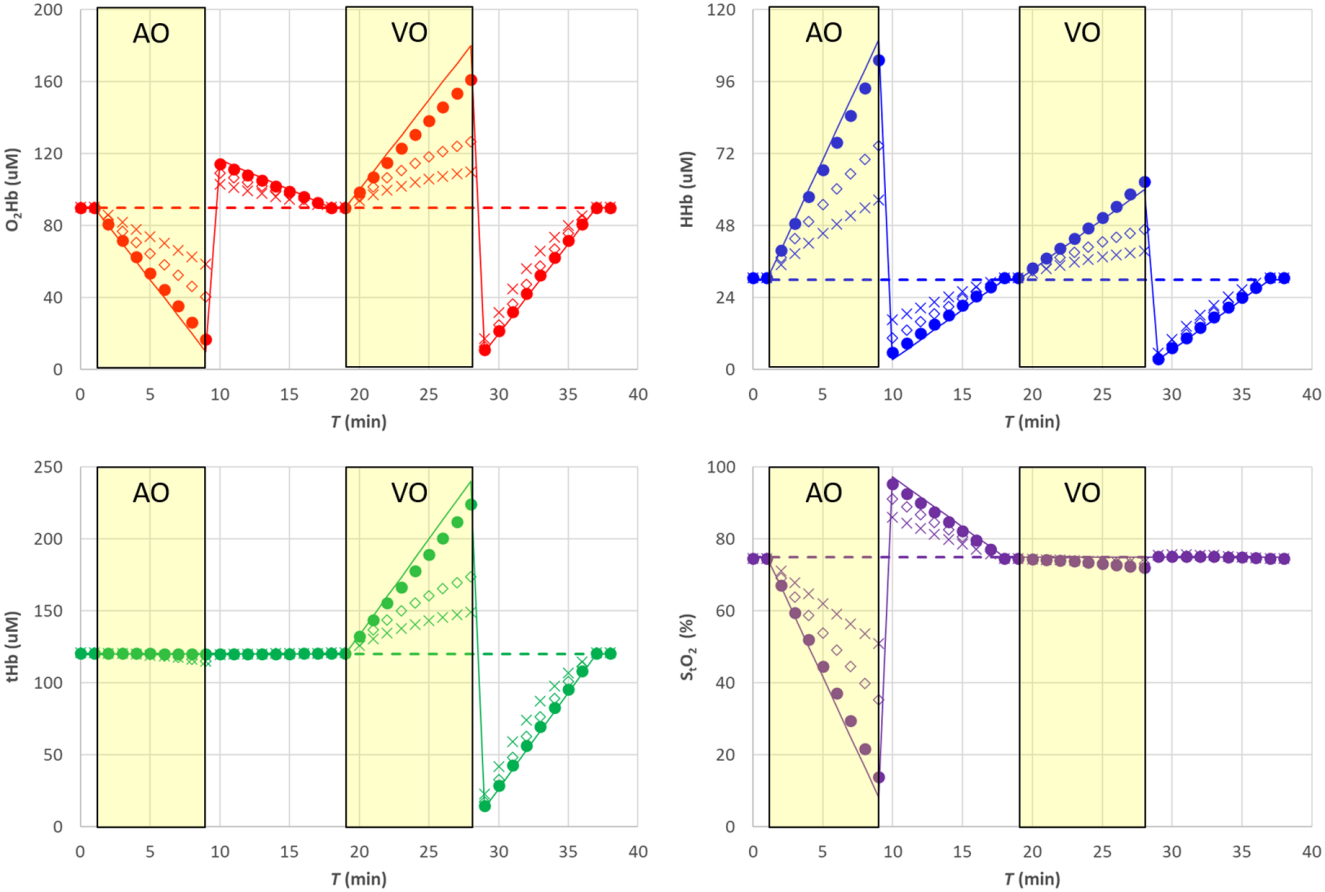
SRS results for a bilayer with thickness of the upper layer (•) 5 mm, (⋄) 7.5 mm, (X) 10 mm. The solid line is the nominal value for the lower layer, the dashed line is the nominal value for the upper layer. The shaded yellow areas represent the AO and VO tests. Changes are applied to the lower layer only.

**Fig. 11:**
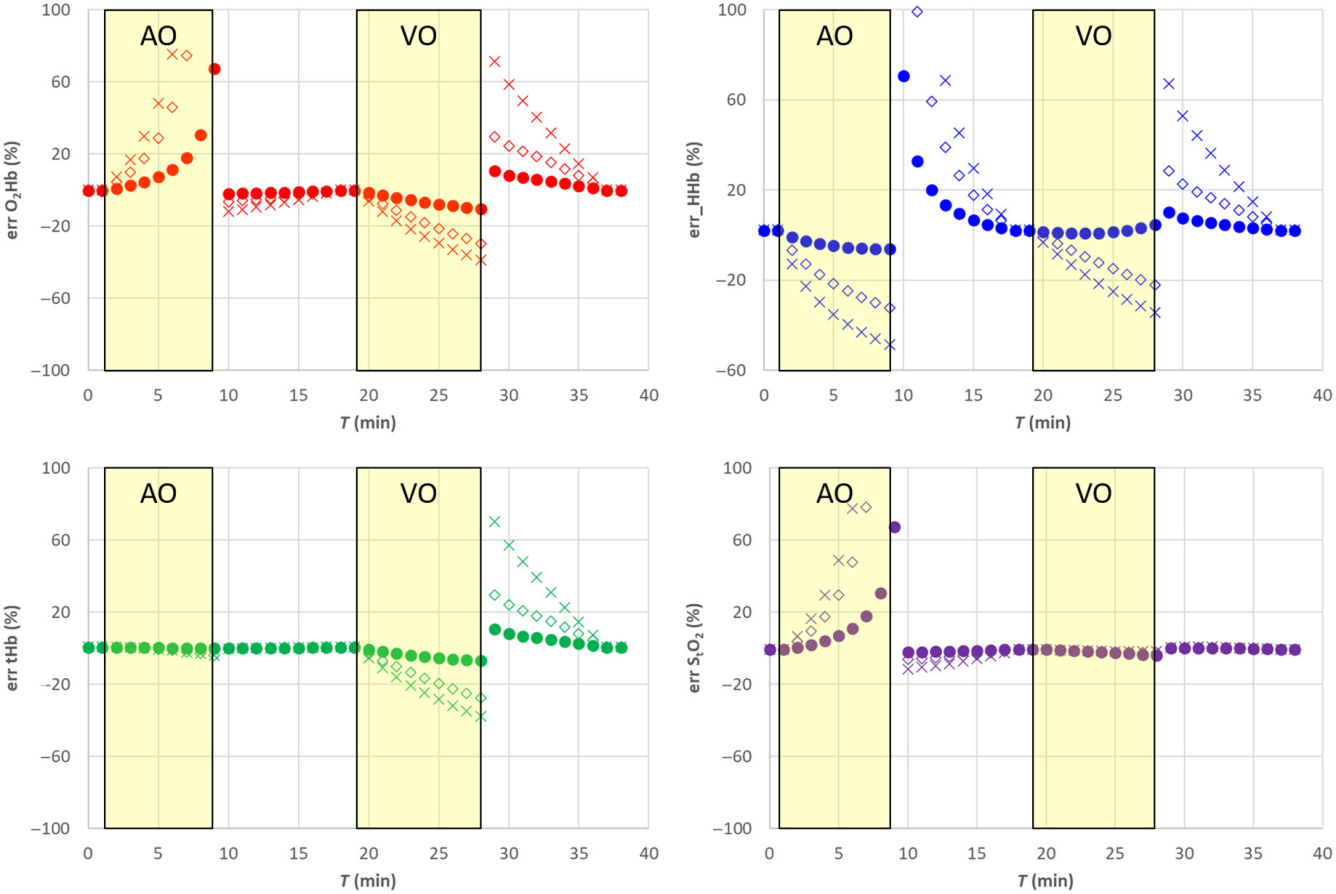
Relative error for the SRS results for a bilayer with thickness of the upper layer (•) 5 mm, (⋄) 7.5 mm, (X) 10 mm. The shaded yellow areas represent the AO and VO tests. Changes are applied to the lower layer only.

**Fig. 12:**
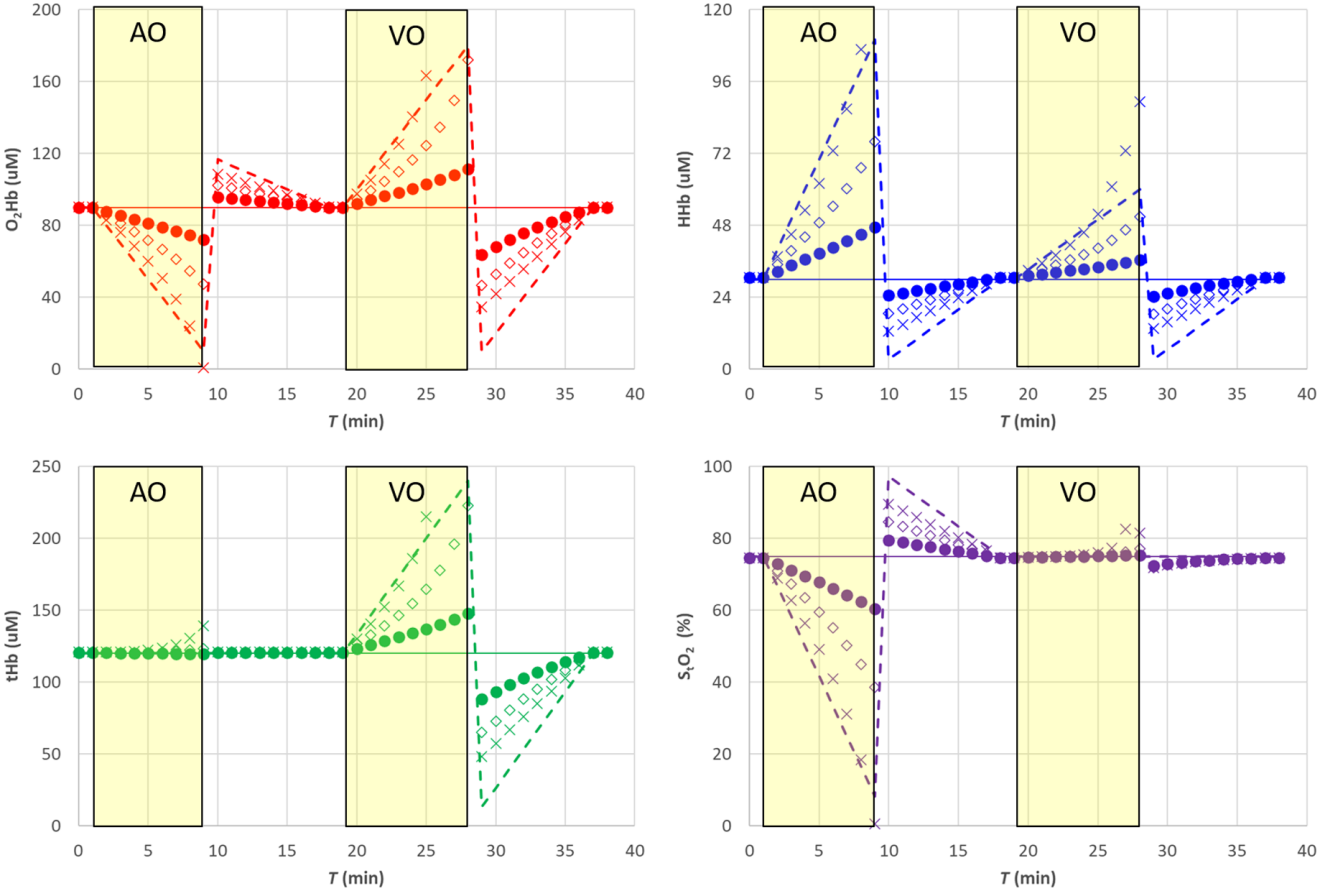
SRS results for a bilayer with thickness of the upper layer (•) 5 mm, (⋄) 7.5 mm, (X) 10 mm. In each panel, the solid line is the nominal value for the lower layer, the dashed line is the nominal value for the upper layer. Changes are applied to the upper layer only.

**Fig. 13:**
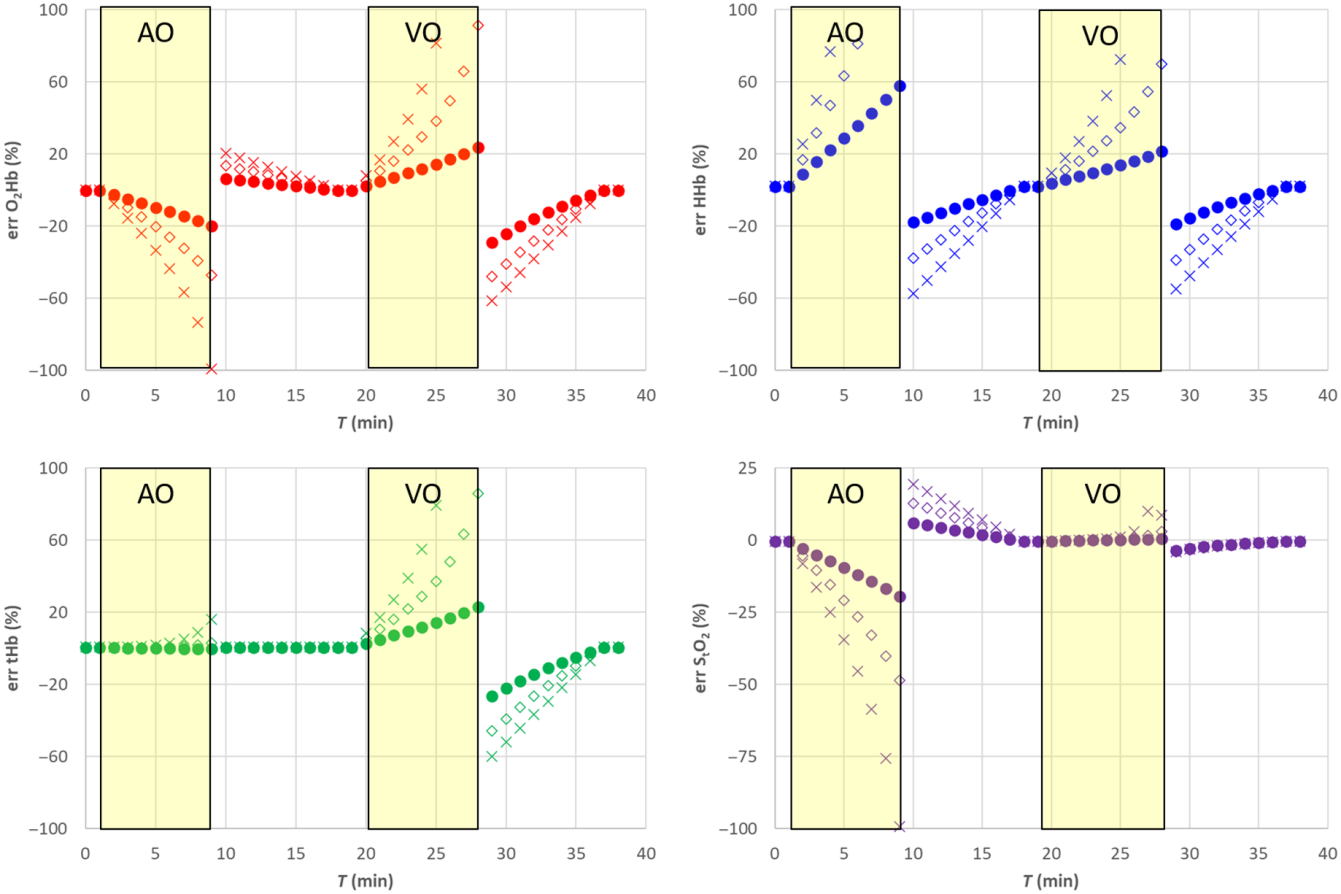
Relative error with respect to the lower layer for the SRS results for a bilayer with thickness of the upper layer (•) 5 mm, (⋄) 7.5 mm, (X) 10 mm. Changes are applied to the upper layer only.

We simulated the presence of a superficial layer with different thicknesses (5, 7.5, and 10 mm). In the first case, hemodynamic changes have been applied only in the deeper layer (see Figs. 10 and 11). In the second case, we applied changes only to the upper layer (see Figs. 12 and 13). In both cases, the simulated reduced scattering coefficient was the same in the two layers, and it was maintained constant in the simulations.

The presence of a superficial layer with constant hemodynamic parameters greatly affects the SRS estimates of the hemodynamic parameters in the lower layer. We observe an overestimation (underestimation) of all hemodynamic parameters when an increase (decrease) with respect to the baseline is present. For a thin (5 mm) upper layer, the error on $S_{t}O_{2}<40\%$ can be lower than 10%, but it significantly increases well over 20% for larger thicknesses.

Conversely, if we apply hemodynamic changes only to the superficial layer, while we are interested in the deeper layer, significant errors appear in $S_{t}O_{2}$ and all other hemodynamic quantities as soon as the superficial layer thickness exceeds 5 mm. In both cases, the simulated data were analyzed using the exact reduced scattering coefficients, therefore the errors were only related to the presence of the superficial layer.

## 5 Discussion

### 5.1 Robustness of the SRS Approach

Under the hypothesis of a semi-infinite homogeneous medium and assuming to exactly know the reduced scattering coefficient, the SRS approach provides quite accurate estimates not only of $S_{t}O_{2}$ but also of $O_{2}\mathrm{Hb}$ and HHb. The use of the ZBC and of the simplifying approximation $\rho \gg z_{s}$ is therefore largely acceptable (as also stated by Ref. 7).

The linear approximation for the spectral dependence of the reduced scattering coefficient can be well tolerated, provided the estimate of parameter h is robust. Unfortunately, reported values for parameter h are diverse, ranging from $4.5\times 10^{-5}\;{\mathrm{nm}}^{-1}$^24^ to $6.3\times 10^{-4}\;{\mathrm{nm}}^{-1}$.^14^ Indeed, as shown in Eq. (8), parameter h depends on parameter b, related to equivalent scatterer size in the tissue. Literature data on parameter b are scarce and extremely variable (see Table 3 in the review paper^25^), therefore this choice is crucial for the accuracy of the SRS approach. The results presented in Sec. 4.3 highlight the need to determine the exact h parameter and the strong error obtained when this parameter is approximated.

Changes in the reduced scattering coefficients may affect the estimate of $S_{t}O_{2}$ especially if unbalanced changes occur at the different wavelengths. If balanced, rather than unbalanced, $\mu_{s}^{\prime }(\lambda )$ changes will occur (e.g., if changes in scatterer density but not in scatter size occur), then the effect on the estimate of $S_{t}O_{2}$ is negligible. Moreover, the results reported in Sec. 4.4 could be associated to inter-subject variability of scattering parameter (±20%). Thus, the error reported in Figs. 3 and 5 (5% for $S_{t}O_{2}>40\%$, and more than 10% for $S_{t}O_{2}<40\%$) could be representative of the mean error performed when multiple subjects are measured.

The strongest limitation of the SRS approach is the assumption on the homogeneity of the medium. The presence of a superficial layer affects the estimates of $S_{t}O_{2}$ independently from which layer the hemodynamic changes occur. *A priori* estimation of the thickness of the upper layer can be easily obtained with a plicometer or even better with ultrasonography. Correction factors for the presence of upper layer with different optical properties could be implemented either by adopting more accurate physical models for photon migration, such as solution of the photon diffusion equation in a two-layer medium,^23^ or applying empirical strategies based on phantom^16^ or *in vivo* measurements.^26^ An indicator of the quality and robustness of the SRS estimate for $S_{t}O_{2}$ in case of tissue heterogeneity can be obtained adopting more than two source detector distances and comparing the absorption in the tissue over multiple distances.^27^ However, we stress that this is not a correction but simply an indicator of measurement quality.

The presence of other tissue chromophores, such as melanin or bilirubin, can further reduce the accuracy of the CW-NIRS SRS method, if not properly considered, such as for pulse oximetry.^28^^,^^29^ These chromophores are typically found in the epidermis; therefore, despite the limited thickness of this layer, all detected photons will be affected. Possible solutions to circumvent the problem of skin pigmentation variance in individuals can be the use of more wavelengths to spectrally discriminate the chromophores^30^^,^^31^ and the use of more complex modeling (e.g., three-layer geometry).^23^

In this study, source detector distances and light wavelengths were chosen in accordance with CW-NIRS SRS devices. Indeed, clinical instruments for CW-NIRS SRS use sensors with different source detector distances tailored to the application: for neonatal monitoring a distance of 25 mm is used,^32^ for adult monitoring a distance of 40 or 50 mm is preferred,^33^^,^^34^ whereas intermediate values are sometimes employed for pediatric population. Increasing source detector distance aims at boosting the photon penetration depth,^35^ therefore improving sensitivity to the deeper tissues, such as brain cortex below scalp and skull or muscle below the adipose tissue.

We highlight that in this work, we have not considered a specific device but simply the SRS method. For this reason, we have not introduced in the simulation any source of instrumental noise. Indeed, noise level in CW-NIRS devices is well tolerable unless extreme conditions (e.g., too high absorption) are considered. Specific simulations should be performed in that case.

The performance of a real SRS device can be ameliorated by adopting specific calibration methods. Companies providing CW-NIRS devices exploit different calibration methods, which are rarely described. As an example, Ref. 36 reports a calibration for CW-NIRS devices with *in vivo* measurements. They consider the tissue $S_{t}O_{2}=S_{a}O_{2}0.25+S_{j}O_{2}\;0.75$, where $S_{a}O_{2}$ is the arterial saturation and $S_{j}O_{2}$ the jugular venous saturation. And they consider a linear factor between the measured and expected saturation: $S_{t}O_{2}=\beta_{0}+\beta_{1}S_{t}O_{2\text{ theor}}$. Those factors, however, cannot be found in literature and make it very hard to reproduce real-life scenarios. Thus, in this work, we focused on the limitations due to the theoretical model, which appear to be not negligible.

Finally, we observe that hemodynamic parameters in this work were tailored to the problem of monitoring skeletal muscle oxidative metabolism. Baseline values for geometrical, optical, and hemodynamic parameters are indeed realistic being reported by many authors (see, for example, Refs. 6, 7, 13). Conversely, the simulated 8 min duration of the vascular occlusions can be considered as an upper limit, useful to test extreme values of the optical and hemodynamic parameters. In case of cerebral oximetry, lower baseline values for $O_{2}\mathrm{Hb}$ and HHb should be used, especially for neonates. However, we do not expect large differences in the results since most of the errors arise from the violation of the homogeneity of tissue structure that is crucial also for cerebral oximetry.

### 5.2 Considerations on the Differential Pathlength Factor

It is worth noting that, in principle, assuming values for k and h would allow one to also get an absolute estimate of the differential pathlength factor (DPF).^37^

The estimate of the DPF is generally taken from the literature (see, for example, Ref. 38) while it could be derived by the SRS approach as well as also noticed by Ref. 39. When $\mu_{\mathrm{eff}}$ is known, assuming both k and h is in fact equivalent to assume $\mu_{s}^{\prime }$, and correspondingly equivalent to assume the DPF. In fact, from the photon diffusion theory^20^ in the ZBC approximation and with $\rho \gg z_{s}$, we have

$$\mathrm{DPF}=\frac{\left\langle l(\rho )\right\rangle }{\rho }=\frac{\frac{\partial A}{\partial \mu_{a}}}{\rho }=\frac{3\mu_{s}^{\prime }\rho }{2(1+\mu_{\mathrm{eff}}\rho )}.$$ (9)

**Fig. 14:**
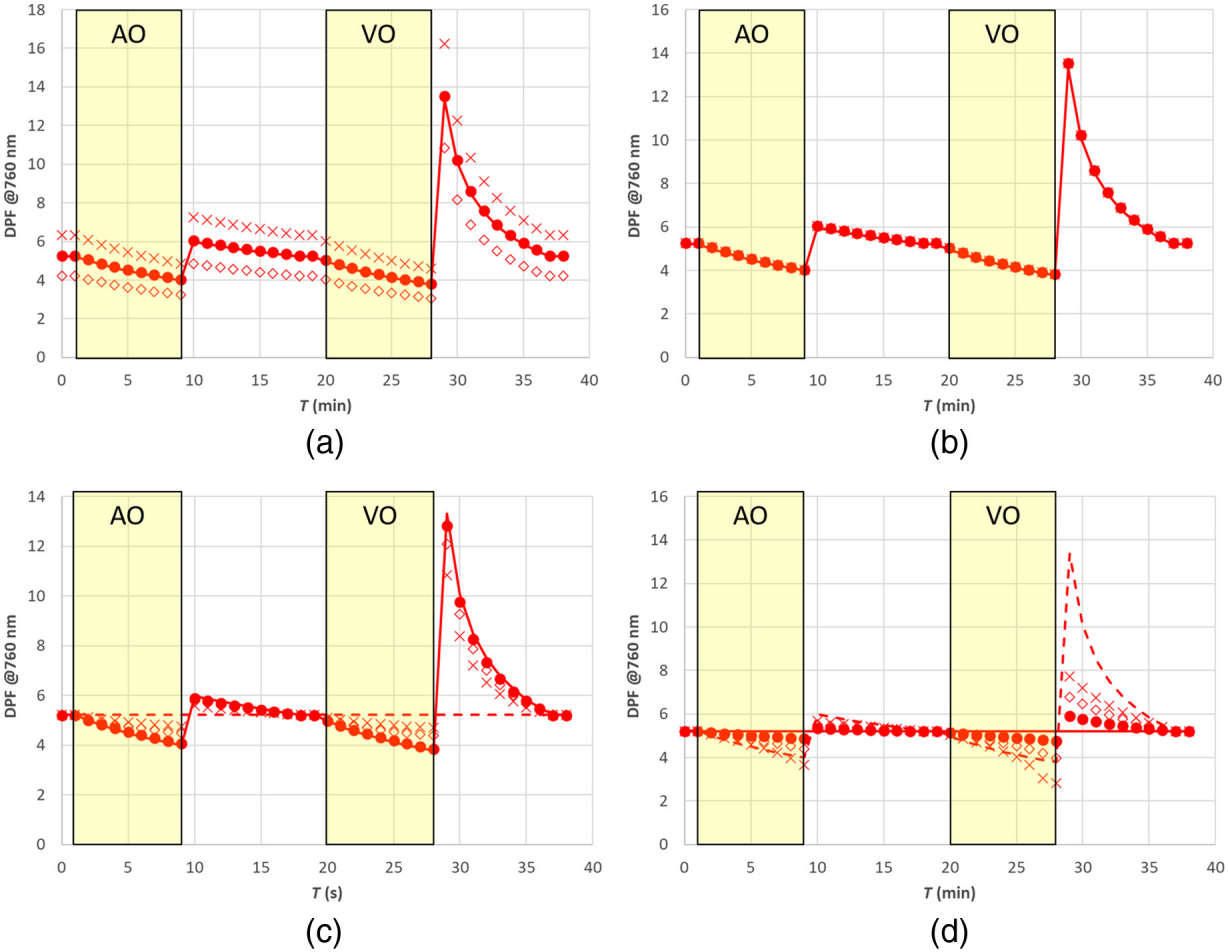
DPF estimate from Eq. (9). (a) Errors in parameter a (⋄) −20%, (X) +20%; (b) errors in parameter b (⋄) −20%, (X) +20%; (c) two-layer medium [thickness of upper layer (•) 5 mm, (⋄) 7.5 mm, (X) 10 mm] with changes in the lower layer and upper layer with constant optical and hemodynamic properties; and (d) two-layer medium [thickness of upper layer (•) 5 mm, (⋄) 7.5 mm, (X) 10 mm] with changes in the upper layer and lower layer with constant optical and hemodynamic properties. Solid lines are exact values.

As expected, the estimate of the DPF is strongly affected by errors in parameter a (or equivalently k) (see Fig. 14 top row), whereas it is less affected by errors in parameter b (or h) (see Fig. 14 middle row). The presence of a superficial layer introduces significant errors in the estimate of DPF (see Fig. 14 bottom row).

### 5.3 Considerations on the Modified Beer Lambert Law

Absolute estimates of $O_{2}\mathrm{Hb}$ and HHb are generally not derived by SRS devices since ${\Delta O}_{2}\mathrm{Hb}$ and ΔHHb (i.e., changes with respect to an arbitrary baseline) are usually obtained by the modified Beer Lambert (MBL) law.^38^ In the MBL law, changes in the absorption coefficient with respect to an arbitrary fixed baseline are derived from changes in light attenuation when using a single source detector distance (the center distance in the SRS scheme) approach for CW-NIRS, provided the parameter the DPF is known^40^

$$\Delta \mu_{a}(\lambda )=\frac{\Delta A(\lambda )}{\left\langle l(\rho )\right\rangle }=\frac{\Delta A(\lambda )}{\rho \mathrm{DPF}(\lambda )},$$ (10)

where ⟨l(ρ)⟩ is the mean photon pathlength in the medium.

From $\Delta \mu_{a}(\lambda )$ at (least) two wavelengths, one can finally get ${\Delta O}_{2}\mathrm{Hb}$ and ΔHHb. Indeed, assuming values for k and h would allow one to also get an absolute estimate of $O_{2}\mathrm{Hb}$ and HHb through Eq. (5).

## 6 Conclusion

In this work, we have recalled the theoretical basis of the CW SRS NIRS approach that is widely used in many commercial tissue oximeters, and we have studied by means of numerical simulations the robustness of the estimate of the hemodynamic parameters. We have shown the effect of errors in the assumption of the reduced scattering coefficient values and of the presence of tissue heterogeneity. We have also reported on the link between SRS parameters k and h and other important parameters in NIRS, such as the DPF, and the Mie parameters a and b. Users should be aware of the limitations intrinsic to the SRS approach. More sophisticated strategies could be considered for CW-NIRS, such as the use of self-calibrating method or the use of multiple short and long distances.^14^

## Code, Data, and Materials Availability

The codes and datasets generated during and analyzed during the current study are available from the corresponding author upon reasonable request.
